# Facial Nerve Hemangioma in the Internal Auditory Canal: A Rare Localization and Therapeutic Challenge

**DOI:** 10.1155/crot/3347034

**Published:** 2026-09-14

**Authors:** Yasmine Elamrani, Mohammed-Amine Salek, Achraf Amine Sbai, Drissia Benfadil, Azzedine Lachkar, Fahd El Ayoubi El Idrissi

**Affiliations:** ^1^ School of Medicine and Pharmacy, Mohammed VI Polytechnic University (UM6P), Benguerir, Morocco, um6p.ma; ^2^ Department of Otolaryngology, Head and Neck Surgery, University Hospital Center Mohamed VI, Oujda, Morocco; ^3^ Faculty of Medicine and Pharmacy, Mohammed First University, Oujda, Morocco; ^4^ Mohammed VI University of Health Sciences (UM6SS), Rabat, Morocco, um6ss.ma

**Keywords:** facial palsy, hemangioma, infratemporal tumors, mastoid approach

## Abstract

**Objective:**

Facial nerve hemangiomas (FNHs) are rare vascular tumors that often mimic other facial nerve disorders, making diagnosis and management challenging. This report highlights a case of FNH in the internal auditory canal (IAC) successfully treated via a translabyrinthine approach.

**Case Presentation:**

A 64‐year‐old female patient with a history of squamous cell carcinoma of the tongue presented with progressive left‐sided peripheral facial paralysis (House–Brackmann Grade IV), associated with ipsilateral hearing loss and vertigo. MRI revealed a nodular lesion in the IAC with imaging features suggestive of a vascular origin. Due to symptom severity, surgical excision was performed using a translabyrinthine approach. Intraoperatively, a vascular lesion compressing but not infiltrating the facial nerve was excised, and histopathology confirmed FNH.

**Results:**

The patient showed significant postoperative improvement, with facial nerve function improving to House–Brackmann Grade II at 6 months.

**Conclusion:**

Early recognition and surgical intervention for symptomatic FNHs can prevent further neurological impairment. The translabyrinthine approach offers effective tumor removal while preserving facial nerve integrity.

## 1. Introduction

Primary tumors of the facial nerve are exceptionally rare, with facial nerve schwannomas being the most frequently encountered. Less common among these are facial nerve hemangiomas (FNHs), which are benign, slow‐growing vascular tumors [1]. These lesions represent approximately 0.7% of intratemporal tumors and account for around 18% of all facial nerve tumors [2].

Magnetic resonance imaging (MRI) is considered the most reliable imaging modality for diagnosing FNHs, as clinical features alone are not pathognomonic [1, 3]. Due to the low prevalence of FNHs, there remains significant debate regarding the most appropriate management strategy [4]. While some experts advocate for delaying surgical intervention until facial nerve function shows marked deterioration, others emphasize early surgical resection to optimize motor function preservation [5].

Here, we present a rare case of a hemangioma located in the internal auditory canal (IAC), successfully treated using a translabyrinthine approach.

## 2. Case Report

We present the case of a 64‐year‐old female patient with a history of squamous cell carcinoma of the lateral tongue margin, previously managed with surgical excision and left triangular neck dissection without adjuvant radiotherapy. She developed progressively worsening left‐sided peripheral facial paralysis over 4 months.

**FIGURE 1:**
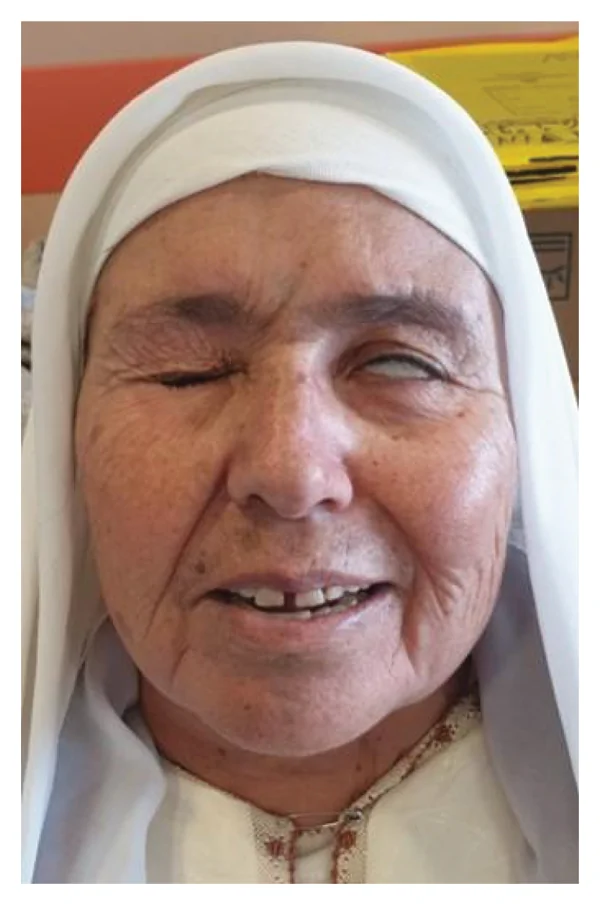
Left peripheral facial paralysis, House–Brackmann Grade IV, with inability to fully close the eye at maximal effort.

**FIGURE 2:**
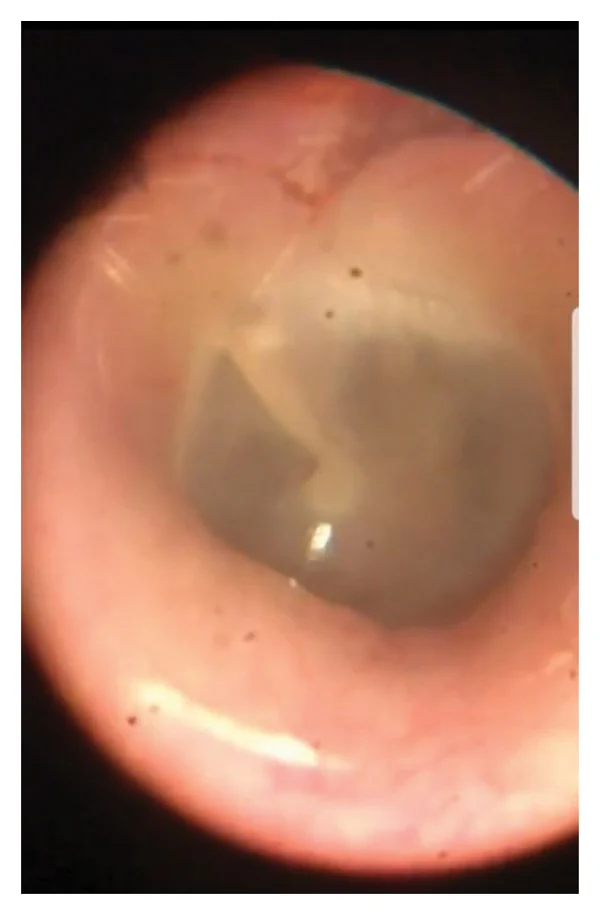
Normal otoscopic examination of the left tympanic membrane, with no visible pathology.

**FIGURE 3:**
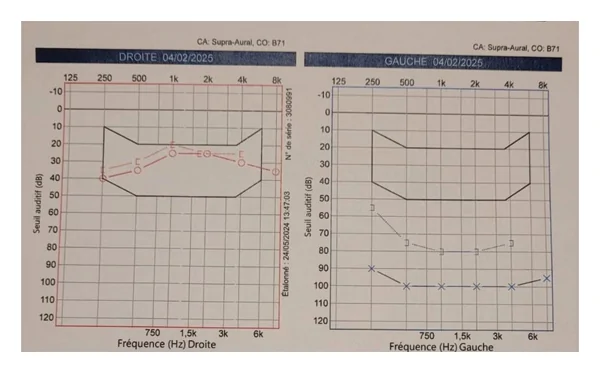
Audiometric evaluation showing profound unilateral sensorineural hearing loss in the left ear.

The patient reported ipsilateral hypoacusis accompanied by persistent rotatory vertigo, described as a continuous sensation of instability. Clinical examination revealed House–Brackmann (HB) Grade IV left‐sided peripheral facial paralysis (Figure 1) with intact facial sensation in all three trigeminal nerve territories and no hypoesthesia in the Ramsay Hunt zone. Otologic examination demonstrated a normal left tympanic membrane with preserved anatomical landmarks (Figure 2). Vestibular assessment identified a left‐sided peripheral vestibular syndrome without signs of central involvement. Pure‐tone audiometry confirmed profound unilateral sensorineural hearing loss in the left ear (Figure 3).

**FIGURE 4:**
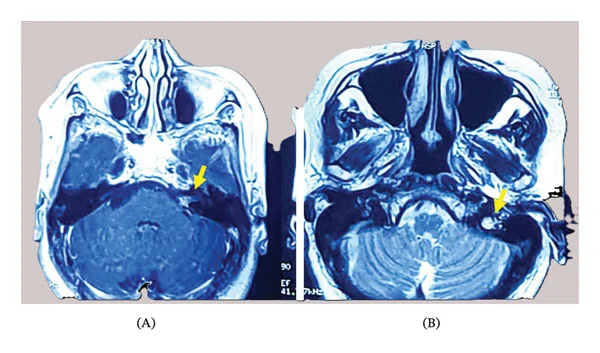
(A) Gadolinium‐enhanced T1‐weighted sequences reveal heterogeneous contrast within the internal auditory canal, highlighting the vascular nature of the facial nerve hemangioma. (B) T2‐weighted sequences demonstrate a hyperintense nodular lesion.

Given the clinical presentation, a lesion involving the cerebellopontine angle or the petrous bone was suspected. Brain MRI revealed a well‐defined unilateral left nodular lesion, measuring less than 1 cm at its largest diameter, situated within a nondilated IAC. On gadolinium‐enhanced T1‐weighted sequences, the lesion exhibited heterogeneous contrast enhancement without expansion of the geniculate ganglion (GG) fossa or extension into the cerebellopontine angle. On T2‐weighted sequences, it appeared hyperintense, suggesting a vascular origin (Figure 4).

Due to the severity of symptoms, including profound hearing loss and moderate facial paralysis, surgical excision was performed, considering the lesion’s small size and the absence of direct facial nerve infiltration. A translabyrinthine approach was chosen, starting with an extended mastoidectomy while preserving anatomical landmarks. Drilling was extended to the lateral, posterior, and superior semicircular canals to access the fundus of the IAC.

**FIGURE 5:**
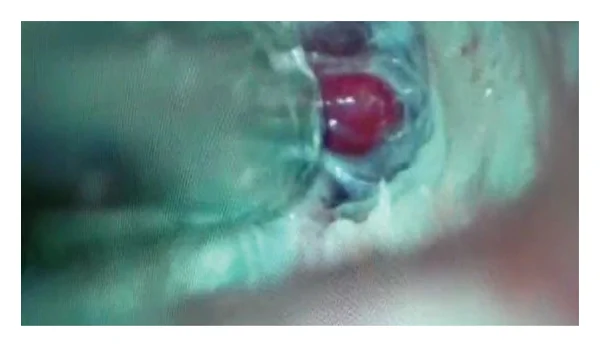
Surgical view after translabyrinthine approach to the internal auditory canal, revealing a facial nerve hemangioma appearing as a bluish, vascular‐appearing lesion.

**FIGURE 6:**
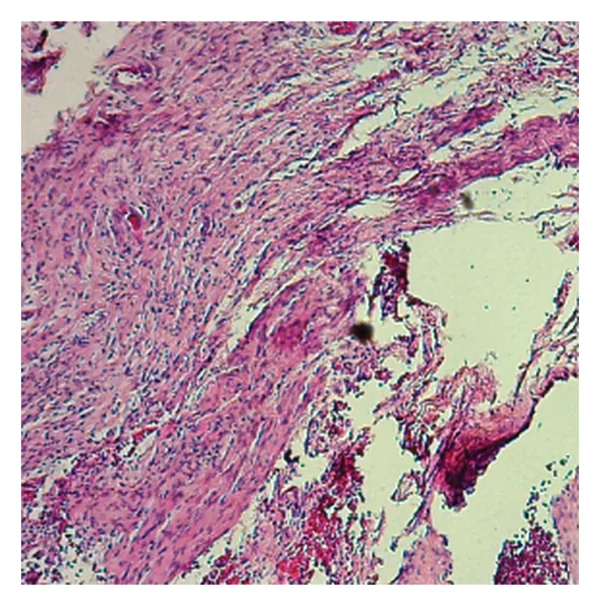
Hematoxylin‐eosin staining revealing vascular structures with a single layer of endothelial cells within the facial nerve perineurium, causing neural compression without evidence of perineural invasion.

Intraoperatively, a bluish, vascular‐appearing lesion was identified, originating from the facial nerve (Figure 5). Careful dissection allowed for complete excision without compromising adjacent structures. Intraoperative evaluation confirmed that the lesion was exerting external compression on the facial nerve without direct invasion, enabling the preservation of nerve integrity. Histopathological analysis confirmed the diagnosis of an FNH (Figure 6).

The postoperative course was favorable, with notable improvement in facial nerve function. At 6 months postsurgery, the patient’s facial paralysis had improved to HB Grade II, demonstrating a positive functional outcome following surgical intervention.

Further long‐term follow‐up is ongoing to assess the durability of functional recovery and to monitor for potential recurrence.

## 3. Discussion

The facial nerve, also referred to as the seventh cranial nerve, plays a critical role in various functions. It governs motor control for facial expressions, facilitates taste sensation in the anterior two‐thirds of the tongue, and provides tactile input to parts of the external ear. Additionally, it supplies parasympathetic innervation to the lacrimal, submandibular, and sublingual glands [4, 6].

FNHs most often originate from the GG, representing around 0.7% of intratemporal vascular abnormalities [1]. They are predominantly reported in individuals between the ages of 30 and 60, with a slight female predominance [3].

The first documented case of a GG hemangioma was reported by Pulec in 1969 [7]. Since then, clinical reports of FNHs remain scarce [6].

FNHs are believed to arise from the venous plexus that surrounds the facial nerve [2]. These lesions can develop along any segment of the facial nerve within the temporal bone; however, they most commonly involve the GG and IAC [8]. Rare cases have also been reported in the second genu of the intratemporal facial nerve, the tympanic portion, and the mastoid segment. This preferential involvement of the GG and IAC may be attributed to the denser distribution of peripheral capillaries in these regions compared to other segments of the facial nerve [3, 9]. Studies have confirmed that the GG contains a higher density of capillary plexuses than other portions of the facial nerve, which may explain its susceptibility to hemangioma formation.

FNHs are slow‐growing tumors that present a wide range of clinical symptoms. These lesions can exert pressure on or infiltrate surrounding structures, leading to significant neurological impairment [10].

A distinguishing feature of FNHs is recurrent or persistent ipsilateral facial nerve paralysis, which is often disproportionate to the tumor’s size [1, 10]. Some patients may experience sudden‐onset facial paralysis or hemifacial spasms [3]. Even small tumors can cause facial nerve dysfunction, suggesting potential direct invasion of nerve fibers [2]. Misdiagnosis as Bell’s palsy is frequent, often resulting in delays in appropriate diagnosis and treatment [1].

Additional symptoms may include facial twitching, sensorineural hearing loss, or cochlear fistula formation as the tumor enlarges [1]. When FNHs occur within the IAC, they typically first manifest as retrocochlear sensorineural hearing loss, with progressive facial nerve dysfunction and vertigo developing over time [8, 10].

Due to the rarity of hemangiomas affecting the tympanic segment of the facial nerve, their clinical characteristics are not well defined [10]. Other reported symptoms include pulsatile tinnitus, vertigo, otalgia, aural bleeding, and, in some instances, a polypoid mass in the external auditory canal. Pulsatile tinnitus and vertigo, though uncommon, may arise due to angiomatous invasion of the cochlea or compression of the vestibular nerve. Conductive hearing loss is most frequently associated with tumor extension into the middle ear [2]. In certain cases, neurotologic symptoms may also be observed [6].

Imaging plays a crucial role in the diagnosis of FNHs, as clinical history alone is often insufficient. A comprehensive evaluation requires a combination of gadolinium‐enhanced MRI and high‐resolution computed tomography (CT).

On high‐resolution CT, characteristic findings of FNHs include lesions with irregular margins and borders, often accompanied by intralesional calcifications forming bone spicules. These calcifications typically produce the distinctive “honeycomb” bone appearance, which is believed to result from a reactive bone remodeling process [1, 2, 4, 8, 9]. In addition, FNHs can lead to irregular expansion of the surrounding bone, further contributing to their radiologic distinctiveness [6, 10].

Distinguishing facial nerve venous malformations from schwannomas is crucial, as the latter typically presents smooth, well‐defined, and scalloped margins, lacking intralesional calcifications. However, facial canal expansion is not a distinguishing feature, as it can also be observed in other pathologies, including schwannomas [3]. Consequently, a comprehensive evaluation of multiple imaging characteristics is necessary to ensure an accurate diagnosis and to differentiate FNHs from other intratemporal tumors.

MRI is the most sensitive imaging technique for diagnosing FNHs [1, 3]. It plays a key role in identifying pathological changes in the nerve and differentiating FNHs from other potential causes of facial nerve palsy, such as parotid gland tumors or intracranial lesions [2]. FNHs typically appear as isointense or hypointense lesions on T1‐weighted imaging relative to adjacent soft tissues [6, 10]. On T2‐weighted sequences, these tumors often exhibit heterogeneous hyperintensity, with variable degrees of enhancement following gadolinium administration [1, 2, 4, 6, 9, 10]. After contrast administration, FNHs present as heterogeneously enhancing masses on T1‐weighted images, often with punctate nonenhancing areas corresponding to bone spicules and intratumoral calcifications [1, 4, 6]. MRI is particularly useful in detecting FNHs located within the IAC, though smaller lesions may still be difficult to identify [6]. Distinguishing FNHs from other tumors in the same region, such as schwannomas, can be challenging, as both entities exhibit similar signal characteristics on T1‐and T2‐weighted sequences and show contrast enhancement [8]. However, anatomical location provides an important distinguishing factor: FNHs are typically confined to the IAC or GG, whereas schwannomas tend to extend along the facial nerve, often involving multiple segments [8]. Additionally, larger FNVMs may demonstrate increased T2 hyperintensity, a feature that may help differentiate them from schwannomas, which generally appear relatively T2 hypointense [3]. Newer MRI sequences can enhance lesion detection and characterization. The conventional two‐dimensional fast spin‐echo (2D FSE) sequence is effective in identifying small tumors; however, the three‐dimensional fast imaging employing steady‐state acquisition (3D FIESTA) sequence has shown superior accuracy in detecting lesions. Despite its advantages, this sequence has not yet been widely investigated in FNH cases [9].

FNHs are uncommon, slow‐growing vascular lesions that arise from perineural capillaries, most frequently in the GG region [4]. Histopathologically, they consist of irregular, dilated vascular channels that lack mitotic activity and internal elastic lamina, with a minimal smooth muscle component. These lesions are often accompanied by intratumoral calcified spicules, likely originating from adjacent bone [1, 3, 4].

The presence of these calcifications has been attributed to localized bone resorption and reactive neonatal bone formation within vascular compartments, a process driven by tumor activity rather than true neoplastic bone production [9]. Although traditionally classified as hemangiomas, histopathological studies indicate that FNHs differ from classic infantile hemangiomas [1, 6]. Immunohistochemical analyses further challenge this classification, as these lesions do not exhibit GLUT1 or Lewis Y antigen expression, markers commonly associated with infantile hemangiomas. Nevertheless, the term “hemangioma” continues to be widely used in clinical practice [1].

FNHs are often misdiagnosed or overlooked due to their rarity and nonspecific clinical manifestations. Distinguishing FNHs from other lesions affecting the facial nerve canal remains challenging, as several conditions can present with similar symptoms. Differential diagnoses include facial nerve schwannomas, as well as other neoplasms such as meningiomas, cholesteatomas, paragangliomas, lymphomas, and metastatic or locally advanced head and neck malignancies, including squamous cell carcinoma [4, 10–12]. Additionally, Bell’s palsy, Hunt syndrome, and perineural spread of parotid malignancies must also be considered [2]. In patients with a history of head and neck squamous cell carcinoma, metastatic disease or perineural tumor spread should be considered in the differential diagnosis of progressive facial nerve palsy. Perineural spread involving the facial nerve has been reported in head and neck malignancies, particularly in parotid tumors, and may occasionally extend toward the IAC [12]. In the present case, the patient’s previous history of tongue squamous cell carcinoma initially raised concern for a malignant etiology. However, MRI demonstrated a small lesion confined to the IAC, without radiological evidence of local recurrence or perineural extension, and histopathological examination ultimately confirmed the diagnosis of FNH.

Facial nerve schwannomas, although less common than vestibulocochlear schwannomas, can occur without significant facial nerve dysfunction and typically appear as well‐defined, rounded lesions on imaging [4]; Among the conditions commonly mistaken for FNHs, Bell’s palsy is a frequent diagnostic challenge. These tumors are often misdiagnosed as Bell’s palsy, particularly when patients show temporary improvement with corticosteroid therapy. However, cases of recurrent paralysis or poor response to steroids should raise suspicion of an underlying pathology, warranting further imaging to confirm the diagnosis [6].

The management of FNHs remains challenging. Given the low incidence of these lesions, no definitive consensus has been established regarding the optimal therapeutic approach [4]. In cases where the tumor exhibits slow growth and remains asymptomatic, a conservative strategy involving regular monitoring and serial imaging may be a viable option to avoid unnecessary interventions [1, 13].

The preferred treatment for FNHs is surgical excision, particularly in cases with progressive symptoms; however, the optimal timing of intervention remains a subject of debate [2, 8, 13]. Early diagnosis and prompt surgical management are recommended, even in the absence of significant facial nerve dysfunction, as early‐stage hemangiomas exert minimal pressure on the nerve, resulting in reduced inflammation and fibrosis. This approach is associated with better postoperative outcomes and a lower risk of recurrence [2, 8, 10]. The choice of surgical technique is influenced by factors such as tumor size, anatomical location, and the degree of preoperative hearing impairment, as well as its impact on facial nerve function [2, 6].

The primary objective of surgical treatment is complete tumor resection, with the selection of the surgical approach—middle fossa, transmastoid, or translabyrinthine—determined by the patient’s hearing status [4, 14]. When facial nerve paralysis exceeds HB Grade III, a middle cranial fossa approach is advised for patients with preserved hearing, whereas a translabyrinthine approach is preferred in cases of profound hearing loss [1, 6]. For tumors affecting the labyrinthine segment or the GG, a transmastoid approach is generally recommended [6, 8].

In certain cases, tumor resection can be performed while maintaining facial nerve integrity, particularly for smaller lesions with minimal compression, limited inflammatory response, and low fibrosis levels. This allows for excision without compromising the motor function of the facial nerve [2, 10]. Recurrence is uncommon following either complete or partial resection [2]. Hemangiomas of larger size, especially those located in the GG, frequently exhibit strong adhesion to the facial nerve, complicating their separation and increasing the difficulty of preserving nerve integrity during surgery [8]. When maintaining nerve integrity is not possible, resection of the affected nerve segment is typically required, followed by reconstruction through either end‐to‐end anastomosis or interposition grafting [1, 2, 10]. In contrast, facial nerve schwannomas tend to grow concentrically around the nerve, often necessitating segmental resection to achieve complete removal [15].

A study conducted by Park et al. reported a unique case of FNH presenting with HB Grade II facial palsy and hemifacial spasm, successfully managed with stereotactic radiosurgery (SRS) as the sole treatment. To the best of their knowledge, this represents the first documented case of FNH treated exclusively with SRS [6].

Given the challenges associated with conventional treatment, Park et al. [6] suggested that SRS may serve as a viable therapeutic alternative in selected cases. This technique delivers highly targeted radiation to the lesion while minimizing damage to surrounding structures. Although not traditionally considered for hemangiomas, SRS has demonstrated efficacy in treating other vascular lesions, including cavernous sinus hemangiomas. The proposed mechanism of action involves inducing inflammation and microvascular collapse, which may contribute to its effectiveness in controlling FNH growth and alleviating associated symptoms [6].

Postoperative results are affected by multiple factors, including the duration of preoperative facial nerve dysfunction and the extent of anatomical preservation during surgery [6]. Research indicates that maintaining facial nerve integrity is associated with improved functional outcomes, especially when the duration of preoperative dysfunction is less than 12 months [6]. However, despite careful surgical techniques, restoring optimal facial nerve function remains a challenge [6]. Additionally, delaying surgical intervention may increase the risk of complications such as cochlear fistula and complete hearing loss [8].

## 4. Conclusion

FNHs are rare vascular lesions, often misdiagnosed due to their nonspecific symptoms. Imaging, particularly MRI with gadolinium enhancement and high‐resolution CT, is essential for accurate diagnosis and differentiation from other facial nerve tumors.

Management remains debated, with conservative monitoring recommended for asymptomatic or slow‐growing cases, while surgical excision is preferred for symptomatic or progressive lesions. Early intervention improves outcomes by minimizing nerve compression, inflammation, and fibrosis. The choice of surgical approach depends on tumor location, size, and hearing status.

When nerve preservation is not feasible, reconstruction through anastomosis or grafting may be necessary. SRS is emerging as a potential alternative for select cases, but further studies are needed. Given the risk of complications such as hearing loss and facial dysfunction, a multidisciplinary approach is essential to optimize patient outcomes.

## Author Contributions

All authors attest that they meet the current ICMJE criteria for authorship.

## Funding

The authors received no specific funding for this work.

## Consent

Informed consent was obtained from the patient.

## Conflicts of Interest

The authors declare no conflicts of interest.

## Data Availability

Image data used to support the findings of this study are included within the article.
